# Comparative expression profiles of host circulating miRNAs in response to *Trichinella spiralis* infection

**DOI:** 10.1186/s13567-020-00758-0

**Published:** 2020-03-11

**Authors:** Xiao Han Ma, Hui Jun Ren, Ruo Yu Peng, Yi Li, Liang Ming

**Affiliations:** 1 Department of Clinical Laboratory, The First Affiliated Hospital of Zhengzhou University, Zhengzhou, 450052 China; 2 Key Clinical Laboratory of Henan Province, Zhengzhou, 450052 China

## Abstract

Trichinellosis is an important food-borne parasitic zoonosis throughout the world. At present, the mechanisms of *Trichinella spiralis* infection remain unclear. Acquiring detailed information on the host-*Trichinella* interaction would be beneficial for the development of new strategies for trichinellosis control. Circulating miRNAs are stably detectable in the blood of humans and animals infected with parasites. Circulating miRNAs might regulate the expression of target genes in pathological responses during infection and might be novel potential biomarkers of parasitic diseases. In the present study, a total of ten differentially expressed circulating mouse miRNAs with |log_2_(fold change)| ≥ 1.0 and FDR < 0.01 were found during *T. spiralis* infection, of which five were upregulated and five were downregulated. GO and KEGG analyses showed that the target genes of the ten miRNAs were enriched in many signalling pathways, especially focal adhesion, MAPK pathway, and so on. The results of qRT-PCR showed that among the five upregulated miRNAs, mmu-miR-467a-3p and mmu-miR-467d-3p expression in mouse serum reached a peak at 30 days post-infection (dpi). The expression of mmu-miR-376b-3p and mmu-miR-664-3p increased significantly at 18 dpi and then decreased at 30 dpi. The expression of mmu-miR-292a-5p gradually decreased from 12 to 30 dpi. Among the 5 downregulated miRNAs, mmu-miR-199a-5p expression was significantly downregulated at 30 dpi, while the expression levels of the other four miRNAs (mmu-miR-455-5p, mmu-miR-125b-5p, mmu-miR-125a-5p, and mmu-miR-615-3p) were significantly lower compared with the control, showing a steady downregulation at different phases of infection. These findings will help to further understand the host-*Trichinella* interaction and provide promising serum biomarkers for trichinellosis.

## Introduction

Trichinellosis is an important food-borne parasitic zoonosis caused by the consumption of raw or undercooked meat containing *Trichinella spiralis* infective larvae. *T. spiralis* can infect a range of animal species, including humans. Human trichinellosis is distributed in 55 countries and is considered an emerging or re-emerging disease [1]. Muscle larvae are released from their capsules after ingestion in the host stomach and then activated into intestinal infective larvae at 0.9 h post-infection (hpi). The infective larvae penetrate into the host intestinal epithelium, where they undergo four moults and mature to adult worms at 30–40 hpi [2]. Female worms produce newborn larvae at 5 days post-infection (dpi), which then penetrate the intestinal epithelium and enter the blood and lymphatic system. These larvae reach the host striated muscle tissue, resulting in the formation of nurse cells at 26 dpi, where the larvae can survive for years [2]. Considering the whole life cycle, it can be seen that entry into host blood circulation and invasion of muscle cells are crucial points for *T. spiralis* infection. Interactions between *T. spiralis* and the host determine whether its infection is established in the host and the degree of clinical symptoms.

microRNAs (miRNAs) are a class of highly conserved (21–23 nucleotides long), endogenous, noncoding RNAs. They participate in physiological and pathological processes by regulating gene expression, such as inflammatory reactions, immune responses, and tumour occurrence [3]. Previous studies have shown that host miRNAs in relevant tissues or cells are dysregulated during infection with parasites, indicating their crucial roles in host responses to pathogen challenge. Functional analyses have shown that some host dysregulated miRNAs, such as miR-20b, are involved in host immune responses against parasitic infection [4]. Moreover, host miRNAs have been demonstrated to alter the host-parasite interaction and thus further influence the infection and development of the parasites [5, 6]. Recent studies found that circulating miRNAs were stably detectable in the blood or body fluids of humans and animals with parasite infection [7, 8]. Hence, they could not only be regarded as key mediators of the host-parasite interaction but could also prove to be potentially useful as novel biomarkers for parasite infection. For instance, miR-233 in the blood serum of mice infected with *Schistosoma* *japonicum* was significantly upregulated and returned to near normal levels after praziquantel treatment, implying that miR-233 may be a potential biomarker for *Schistosoma* infection [9]. Since parasite-specific antibodies cannot be detected during the early stage of infection, there are few biomarkers with high sensitivity and high specificity for early infection. Identification of circulating miRNAs of hosts infected with parasites will provide new approaches for biomarkers of early infection.

*T. spiralis* species-associated miRNAs have recently been identified by Solexa deep sequencing and biological analyses [10], providing a possibility to better understand their roles in the establishment of infection, growth and development, and host-*Trichinella* interactions [11]. However, host circulating miRNAs associated with *T. spiralis* infection have not been profiled yet. In the present study, ten host circulating miRNAs were dysregulated during *T. spiralis* infection. KEGG analysis showed that several of them participate in the MAPK signalling pathway, Focal adhesion, and so on. These findings will help us to further understand the host-*Trichinella* interaction and identify highly effective and specific biomarkers and prophylactic vaccines.

## Materials and methods

### Ethics statement

All animal experiments in this study were employed in strict accordance with the Animal Ethics Procedures and Guidelines (People’s Republic of China). All animal experimental procedures reported herein were approved by the Ethics Committee of Scientific Research and Clinical Trial of the First Affiliated Hospital of Zhengzhou University (Permit No.: 2019-KY-267).

### Parasites and animals

In this study, the *T. spiralis* isolate strain ISS534 was obtained from a domestic pig in Nanyang city of Henan Province, China. The isolate was maintained in Kunming female mice. Specific pathogen-free BALB/c mice were obtained from the Experimental Animal Center of Henan Province. These mice were provided access to distilled water and sterilized food.

### Collection of mouse serum post-infection

*T. spiralis* muscle larvae were obtained from muscles of Kunming mice using standard pepsin/hydrochloric acid artificial digestion [12]. Sixty BALB/c mice (6–8 weeks old) were randomly and equally divided into two groups. Each mouse in the experimental group (E) was orally inoculated with approximately 500 muscle larvae. At the same time, mice inoculated with saline solution were used as the control (C). Whole blood was drawn from the orbital sinus of each mouse at 30 dpi. The blood samples were allowed to stand at 4 °C for 1 h, and serum (approximately 200 μL) from each mouse was separated by centrifugation at 3500 × *g* for 10 min at 4 °C. The serum of ten mice in each group was randomly pooled as one biological replicate, and each group had three biological replicates. Serum samples were stored at −80 °C to be utilized for RNA extraction.

### Small RNA sequencing

Total RNA was extracted from serum samples using RNAiso Blood reagent (Takara) according to the manufacturer’s instructions. Total RNA purity and concentration were estimated using a Nanodrop 2000 (Thermo Fisher Scientific, USA) at 260 nm and 280 nm. RNA fragments were separated using polyacrylamide gel electrophoresis (PAGE), and small RNAs with lengths of 18–30 nt were recovered. Then, 3′ and 5′ adaptors were ligated to the ends of recovered small RNAs as PCR primers. The cDNAs were obtained and amplified by reverse transcription PCR. The PCR products were purified using PAGE and then dissolved in EB solution to generate the sequencing libraries. The yield and quality of the libraries were evaluated using an Agilent 2100 Bioanalyzer and the StepOnePlus^TM^ Real-Time PCR System (ABI). The cDNA libraries were sequenced by the HiSeq 2000 platform (Illumina, USA), and raw reads were generated.

### Analysis of sequencing data

Raw reads obtained by HiSeq sequencing were filtered through the following steps: (1) removing low-quality reads; (2) removing N reads with proportions greater than 10% (N means unable to determine the base information); and (3) removing reads with 5′ primer contaminants, reads with poly A, reads without 3′ primer or the insert fragments, and reads shorter than 18 nt. Then, clean reads were analysed for their sequence types, sequence number, and length distribution. The clean reads were mapped to the reference mouse genome (NCBI: GCA_000001635.8) using SOAP and Bowtie software without any mismatch to remove contaminant reads from the *Trichinella* transcriptome. Reads of rRNA, scRNA, snoRNA, snRNA, and tRNA were removed by mapping clean reads to the GenBank database [13] and Rfam [14]. The remaining reads were mapped to the miRBase v21.0 database [15] to obtain known miRNAs. The novel miRNAs were identified by using miRDeep2 software as described previously [16].

### Analysis of differentially expressed miRNAs

To identify the differentially expressed circulating miRNAs in mice infected with *T. spiralis*, the expression of miRNAs in each library was normalized to the number of transcripts per million (TPM) [17], and differential expression analysis between the E and C groups was performed using Student’s *t* test. *P* values were adjusted using the Benjamini & Hochberg method, and a corrected *P* value (FDR) of 0.01 was considered statistically significant. Then, the normalized expression values were used to calculate the fold change of each miRNA in the two groups using the formula fold changes = experimental group/control group. The miRNAs with |log_2_(fold change)| ≥ 1.0 and FDR < 0.01 were defined as differentially expressed between the two groups.

### qRT-PCR analysis of differentially expressed miRNAs

To further validate the sequencing results, all ten differentially expressed miRNAs (mmu-miR-467a-3p, mmu-miR-467d-3p, mmu-miR-292a-5p, mmu-miR-376b-3p, mmu-miR-664-3p, mmu-miR-455-5p, mmu-miR-125b-5p, mmu-miR-125a-5p, mmu-miR-615-3p, and mmu-miR-199a-5p) were detected in mouse serum at 30 dpi using qRT-PCR. The specific stem-loop RT primers for each miRNA were designed using Primer 5.0 software (Table 1). The U6 snRNA was used as a reference gene to normalize gene expression. A total of 180 BALB/c mice were randomly and equally divided into the E and C groups. Each mouse in the E group (90 mice) was orally inoculated with approximately 500 ML, and in the C group, the mice were inoculated with saline solution. At 30 dpi, the mice were subjected to serum collection via orbital sinus bleeding, and then they were sacrificed. The pooled serum from ten mice was used as one sample, and thus, every group had 9 mixed serum samples. Total RNA was extracted from each serum sample using RNAiso Blood reagent (Takara) and was reverse-transcribed using PrimeScript RT reagent Kit with gDNA Eraser (Takara). qRT-PCR was performed using TB Green Premix Ex Ta II (Takara). The PCRs in total volume of 10 µL were analysed on Roche LightCycler^®^ 480 II with the following cycling parameters: 1 cycle at 95 °C for 30 s, followed by 40 cycles of amplification at 95 °C for 5 s and 60 °C for 30 s. Relative expression levels of miRNAs were normalized to U6 levels and calculated using the 2^−ΔΔCt^ method [18]. The results are shown as the mean ± SD. For qRT-PCR, each sample was analysed in technical triplicates.

**Table 1:** **Primers used for qRT-PCR to determine miRNA expression**

Name	MiRNA sequence	RT primer 5′–3′	QRT-PCR F primer 5′–3′
mmu-miR-467a-3p	CAUAUACAUACACACACCUACA	GTCGTATCCAGTGCAGGGTCCGAGGTATTCGCACTGGATACGACTGTAGG	GCGCGCATATACATACACACA
mmu-miR-467d-3p	AUAUACAUACACACACCUACAC	GTCGTATCCAGTGCAGGGTCCGAGGTATTCGCACTGGATACGACGTGTAG	CGCGCGATATACATACACACAC
mmu-miR-292a-5p	ACUCAAACUGGGGGCUCUUUUG	GTCGTATCCAGTGCAGGGTCCGAGGTATTCGCACTGGATACGACCAAAAG	GCGACTCAAACTGGGGGCT
mmu-miR-376b-3p	AUCAUAGAGGAACAUCCACUU	GTCGTATCCAGTGCAGGGTCCGAGGTATTCGCACTGGATACGACAAGTGG	CGCGCGATCATAGAGGAACAT
mmu-miR-664-3p	UAUUCAUUUACUCCCCAGCCUA	GTCGTATCCAGTGCAGGGTCCGAGGTATTCGCACTGGATACGACTAGGCT	GCGCGTATTCATTTACTCCCC
mmu-miR-455-5p	UAUGUGCCUUUGGACUACAUCG	GTCGTATCCAGTGCAGGGTCCGAGGTATTCGCACTGGATACGACCGATGT	CGCGTATGTGCCTTTGGACT
mmu-miR-125b-5p	UCCCUGAGACCCUAACUUGUGA	GTCGTATCCAGTGCAGGGTCCGAGGTATTCGCACTGGATACGACTCACAA	CGCGTCCCTGAGACCCTAAC
mmu-miR-125a-5p	UCCCUGAGACCCUUUAACCUGUGA	GTCGTATCCAGTGCAGGGTCCGAGGTATTCGCACTGGATACGACTCACAG	GCGTCCCTGAGACCCTTTAAC
mmu-miR-615-3p	UCCGAGCCUGGGUCUCCCUCUU	GTCGTATCCAGTGCAGGGTCCGAGGTATTCGCACTGGATACGACAAGAGG	CGTCCGAGCCTGGGTCTC
mmu-miR-199a-5p	CCCAGUGUUCAGACUACCUGUUC	GTCGTATCCAGTGCAGGGTCCGAGGTATTCGCACTGGATACGACGAACAG	CGCGCCCAGTGTTCAGACTAC

qRT-PCR Universal R primer 5′–3′ AGTGCAGGGTCCGAGGTATT

Moreover, the expression changes of these miRNAs were also analysed at 12 and 18 dpi using qRT-PCR. At every time point, every group (E or C group) had 9 mixed serum samples. The method of serum collection was the same as that at 30 dpi. Then, the serum samples were used to evaluate the relative expression of these miRNAs at 12 and 18 dpi.

### miRNA target prediction and function analysis

Target genes of miRNAs were identified using the four prediction programmes TargetScan [19], miRanda [20], PITA [21], and RNAhybrid [22], and only miRNA target genes from the common predicted results were considered for further analysis. Target genes of the differentially expressed miRNAs were used in Gene Ontology (GO) and Kyoto Encyclopedia of Genes and Genomes (KEGG) pathway enrichment analyses using the DAVID database [23]. The *P* value was calculated based on Fisher’s exact test, and *P* < 0.05 was considered significant [23].

## Results

### Characteristics of small RNA sequencing

**Figure 1:**
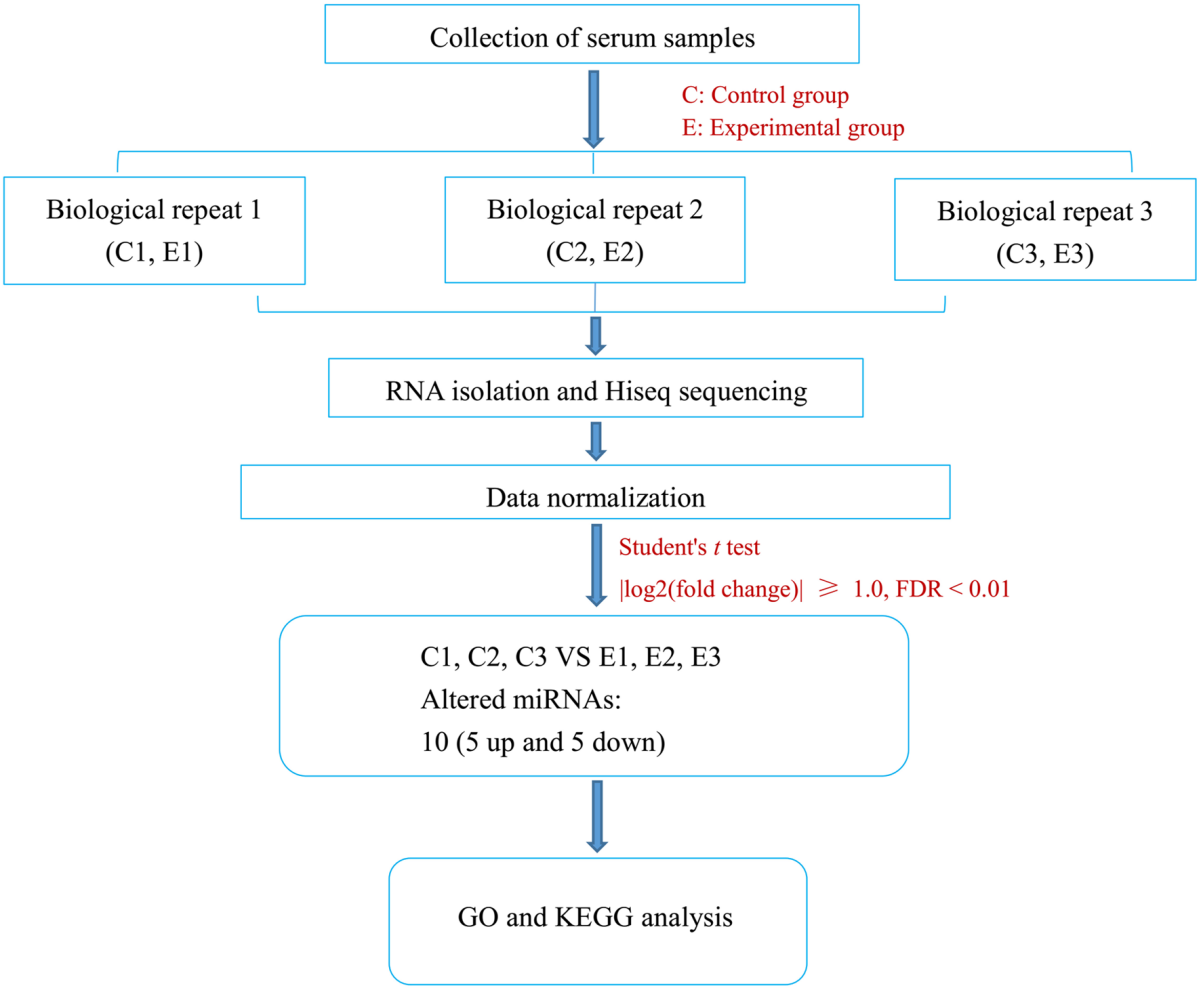
**Workflow of the identification of differentially expressed miRNAs in the serum of mice infected with*T. spiralis***. The serum samples (biological repeats) were collected from uninfected mice (control group, C) and infected mice (experimental group, E). Total RNA was extracted from samples, and HiSeq sequencing and bioinformatics analyses were performed to identify the differentially expressed miRNAs in mouse serum after *T. spiralis* infection. Then, GO and KEGG analyses were further used to predict their functions.

**Figure 2:**
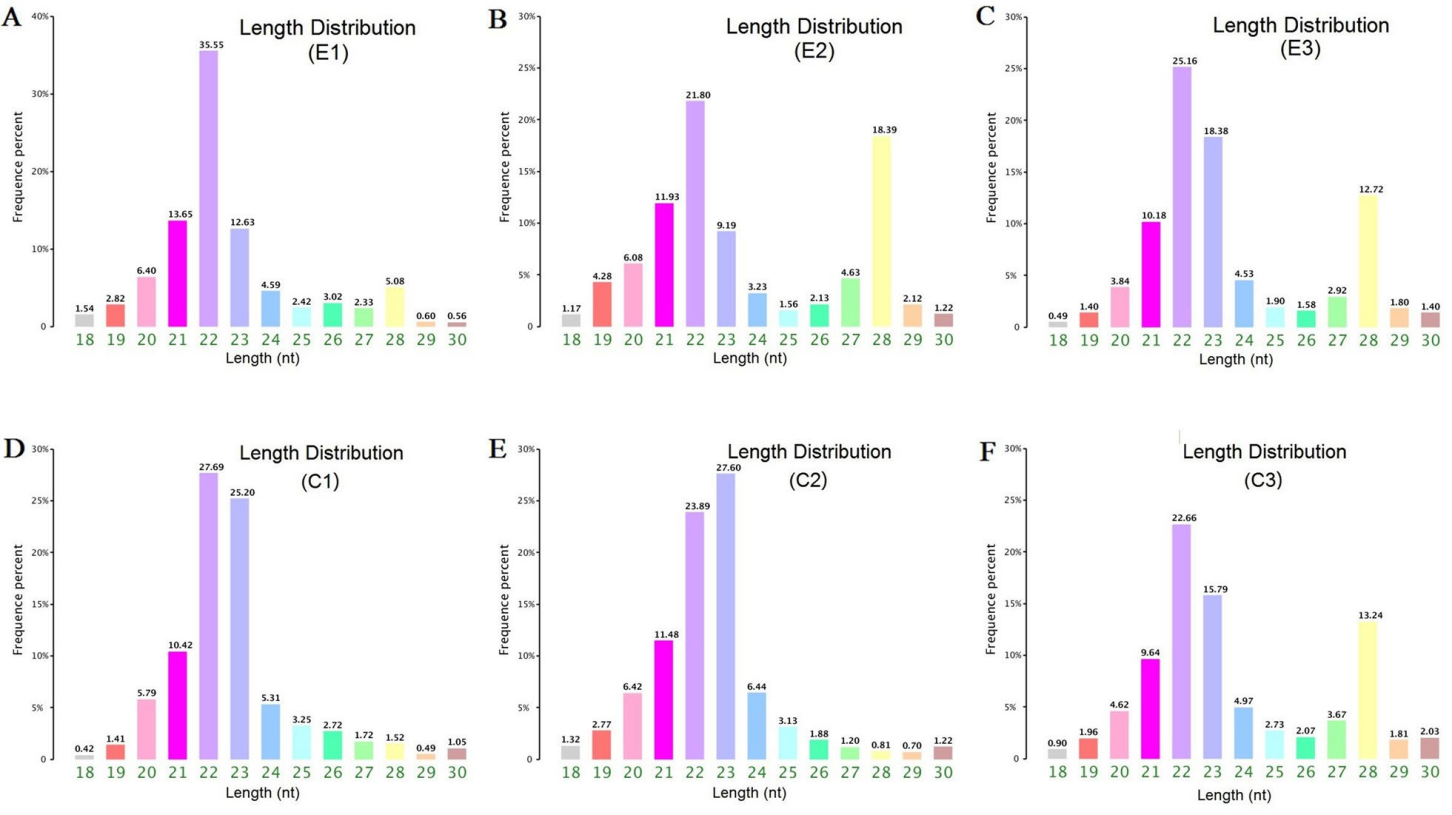
**Length distribution of small RNAs in the serum of mice infected or uninfected with*T. spiralis***. **A**, **B**, **C** Three infected serum samples at 30 dpi. **D**, **E**, **F** Three uninfected serum samples. The length of miRNAs in six mouse serum samples mainly ranged from 21 to 23 nt, which was consistent with the features of miRNAs.

**Figure 3:**
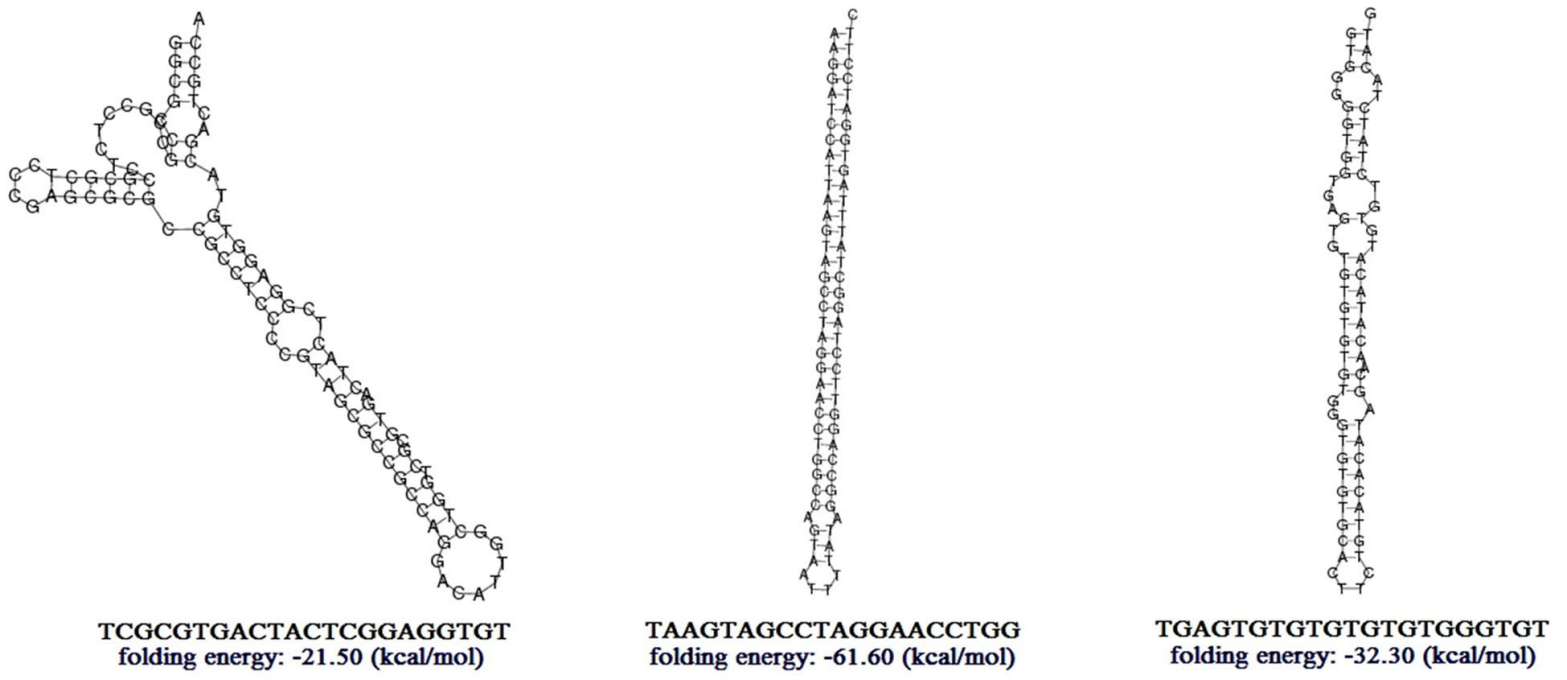
**The secondary structures of several predicted novel miRNA precursors and the corresponding mature sequences.**

In the present study, the differentially expressed miRNAs in the blood serum of mice infected with *T. spiralis* at 30 dpi were compared with those in uninfected mice. The workflow of this study is shown in Figure 1. After removing low-quality reads and linker sequences, high-quality clean reads of six libraries were obtained, as shown in Additional file 1. The length of small RNAs mainly ranged from 21 to 23 nt (Figure 2), which was consistent with miRNA length. Small RNAs of the six libraries were annotated after mapping to the GenBank database and Rfam of *T. spiralis*. In addition to miRNAs, other small RNAs, including rRNAs, scRNAs, snoRNAs, snRNAs, and tRNAs, were also detected, as shown in Additional file 2. After alignment to the miRBase (version 21.0) database, known miRNAs were then obtained for further analysis (see Additional file 3). In addition, novel miRNAs were identified, and their sequences are displayed in Additional file 4. The secondary structures of novel miRNA precursors were predicted, several of which are shown in Figure 3.

### Differentially expressed circulating miRNAs of mice infected with *T. spiralis*

To investigate expression changes of host miRNAs after *T. spiralis* infection, mouse miRNAs in the infected sera were compared with those of the controls. The expression levels of miRNAs in the *T. spiralis*-infected group (three biological repeats) were normalized, and the same analysis was applied to the control group. The relative expression levels of miRNAs are shown in Additional file 5. Subsequently, miRNAs with |log_2_ (fold change)| ≥ 1.0 and FDR < 0.01 were considered differentially expressed. The results showed that ten differentially expressed known miRNAs were found in the infected mouse sera. Among them, 5 miRNAs (mmu-miR-467a-3p, mmu-miR-467d-3p, mmu-miR-292a-5p, mmu-miR-376b-3p, and mmu-miR-664-3p) were upregulated and 5 miRNAs (mmu-miR-455-5p, mmu-miR-125b-5p, mmu-miR-125a-5p, mmu-miR-615-3p, and mmu-miR-199a-5p) were significantly downregulated (Table 2). No differentially expressed novel miRNAs were identified. Cluster analysis was performed to investigate the expression patterns of all the altered miRNAs based on the |log_2_ (E/C)| and *P* value.

**Table 2:** **A subset of putative target genes and GO terms of altered host circulating miRNAs after*T. spiralis*infection**

Name	Length	log_2_ (fold change)	FDR	GO term	Target gene
miR-467a-3p	22	11.99	0.005113502	GO:0005515 protein binding	Zinc finger protein 516 (Zfp516)
				GO:0046872 metal ion binding	Nuclear factor of activated T cells
				GO:0005178 integrin binding	Cytoplasmic, calcineurin dependent 2 (Nfatc2)
				GO:0016881 acid-amino acid ligase activity	TGF-beta activated kinase 1/MAP3K7 binding protein 3 (Tab 3)
miR-467d-3p	22	11.99	0.0051135	GO:0005515 protein binding GO:0008134 transcription factor binding GO:0005178 integrin binding GO:0016881 acid-amino acid ligase activity	Vascular endothelial growth factor A (Vegfa) activating transcription factor 2 (Atf2) transcription factor 3 (Tcf3)
miR-292a-5p	22	5.78	1.55E − 07	GO:0008092 cytoskeletal protein binding	Cadherin-related family member 1 (Cdhr1)
				GO:0019899 enzyme binding	Mast cell immunoglobulin like receptor 1 (Milr1)
				GO:0005515 protein binding	Tight junction protein 3 (Tjp3)
				GO:0008168 methyltransferase activity	Protein kinase C and casein kinase substrate in neurons 3 (Pacsin3)
miR-376b-3p	21	5.05	0.009988095	GO:0004672 protein kinase activity	Laminin, alpha 5 (Lama5)
				GO:0016740 transferase activity	Dual specificity phosphatase 22 (Dusp22)
				GO:0042802 identical protein binding	Membrane associated tyrosine/threonine 1 (Pkmyt1) protein kinase
				GO:0005216 ion channel activity	calcium/calmodulin-dependent protein kinase IV (Camk4)
miR-664-3p	22	3.56	0.005113502	GO:0005229 intracellular calcium activated chloride channel activity	Protein kinase C substrate 80 K-H (Prkcsh)
				GO:0008237 metallopeptidase activity GO:0005509 calcium ion binding	
miR-455-5p	22	−3.04	0.009449003	GO:0042605 peptide antigen binding	Calponin 2 (Cnn2)
				GO:0030881 beta-2-microglobulin binding	Phosphatase and actin regulator 1 (Phactr1)
				GO:0042608 T cell receptor binding	Protein kinase C, beta (Prkcb)
				GO:0004674 protein serine/threonine kinase activity	Alpha-kinase 1 (Alpk1)
				GO:0008235 metalloexopeptidase activity	
miR-125b-5p	22	−3.11	0.005545304	GO:0005515 protein binding	Tight junction associated protein 1 (Tjap1)
				GO:0004672 protein kinase activity	Interferon regulatory factor 1 (Irf1)
				GO:0016740 transferase activity	Chemokine (C–C motif) receptor 8 (Ccr8); CD247 antigen (Cd247)
				GO:0004222 metalloendopeptidase activity	MAP/microtubule affinity regulating kinase 2 (Mark2)
miR-125a-5p	24	−3.21	0.005113502	GO:0005515 protein binding	Tubulin, gamma complex associated protein 6 (Tubgcp6)
				GO:0003779 actin binding	Interleukin 6 signal transducer (Il6st)
				GO:0004674 protein serine/threonine kinase activity	Protein kinase C, zeta (Prkcz)
				GO:0008237 metallopeptidase activity	Chemokine (C–C motif) receptor 9 (Ccr9)
miR-615-3p	22	−3.369881789	0.005545304	GO:0005515 protein binding GO:0004712 protein serine/threonine/tyrosine kinase activity GO:0008092 cytoskeletal protein binding GO:0016787 hydrolase activity	Zinc finger protein 592 (Zfp592) CD40 antigen (Cd40) Dual specificity Phosphatase 6 (Dusp6) Dynamin binding protein (Dnmbp)
miR-199a-5p	23	−3.557273923	0.003171509	GO:0019901 protein kinase binding GO:0050839 cell adhesion molecule binding GO:0051011 microtubule minus-end binding GO:0008233 peptidase activity	Protein kinase N1 (Pkn1) Phosphodiesterase 10A (Pde10a) Junction plakoglobin (Jup) Protein kinase C, zeta (Prkcz)

### Verification of differentially expressed miRNAs by qRT-PCR

**Figure 4:**
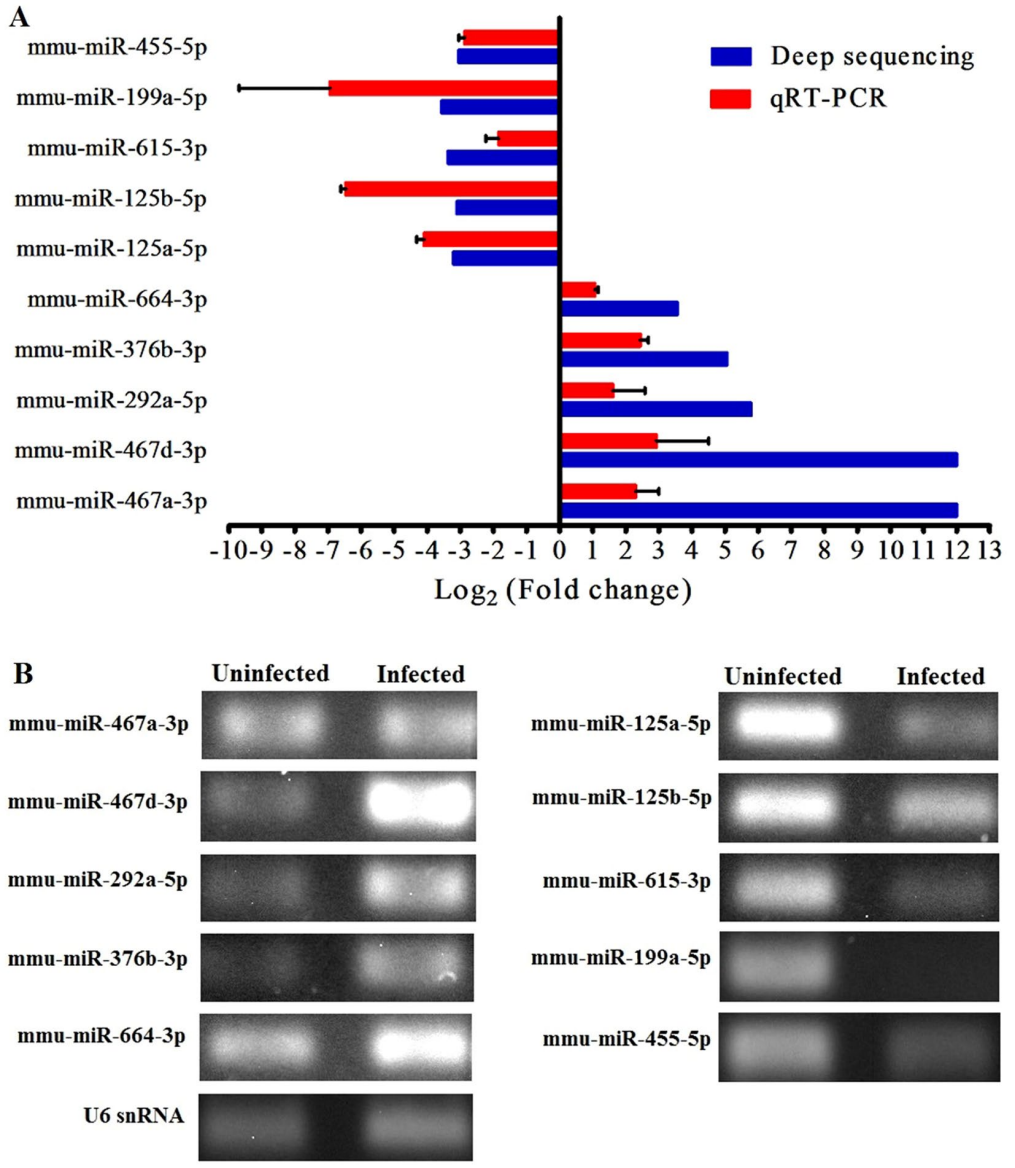
**Verification of the ten differentially expressed mouse circulating miRNAs by qRT-PCR**. The expression levels of all ten differentially expressed miRNAs, which were identified from high-throughput sequencing and bioinformatics analyses, were analysed using qRT-PCR to validate the sequencing results. **A** The relative expression levels by qRT-PCR of the ten miRNAs in the sera of mice infected with *T. spiralis*. Total RNA was isolated from infected mouse serum at 30 dpi and subjected to qRT-PCR as described in “Materials and methods”. Expression levels were normalized to the U6 snRNA levels, and relative expression values were calculated as follows: fold change = 2^−ΔΔCt^. The final relative expression is presented as log_2_ (fold change) (mean ± SD), which is a log_2_ transformation of the fold change. Red and blue columns indicate the results of qRT-PCR and high-throughput sequencing, respectively. **B** The qRT-PCR products of the ten miRNAs were confirmed by agarose gel electrophoresis.

To further verify the sequencing data, all ten differentially expressed miRNAs were analysed by qRT-PCR. The expression trends obtained for the altered miRNAs were consistent with the original HiSeq sequencing results. There was an increase in the expression of mmu-miR-467a-3p, mmu-miR-467d-3p, mmu-miR-292a-5p, mmu-miR-376b-3p and mmu-miR-664-3p and a reduction in the expression of mmu-miR-455-5p, mmu-miR-125b-5p, mmu-miR-125a-5p, mmu-miR-615-3p, and mmu-miR-199a-5p in the serum of mice infected with *T. spiralis* (Figure 4).

### Prediction of miRNA target genes and functional enrichment

**Figure 5:**
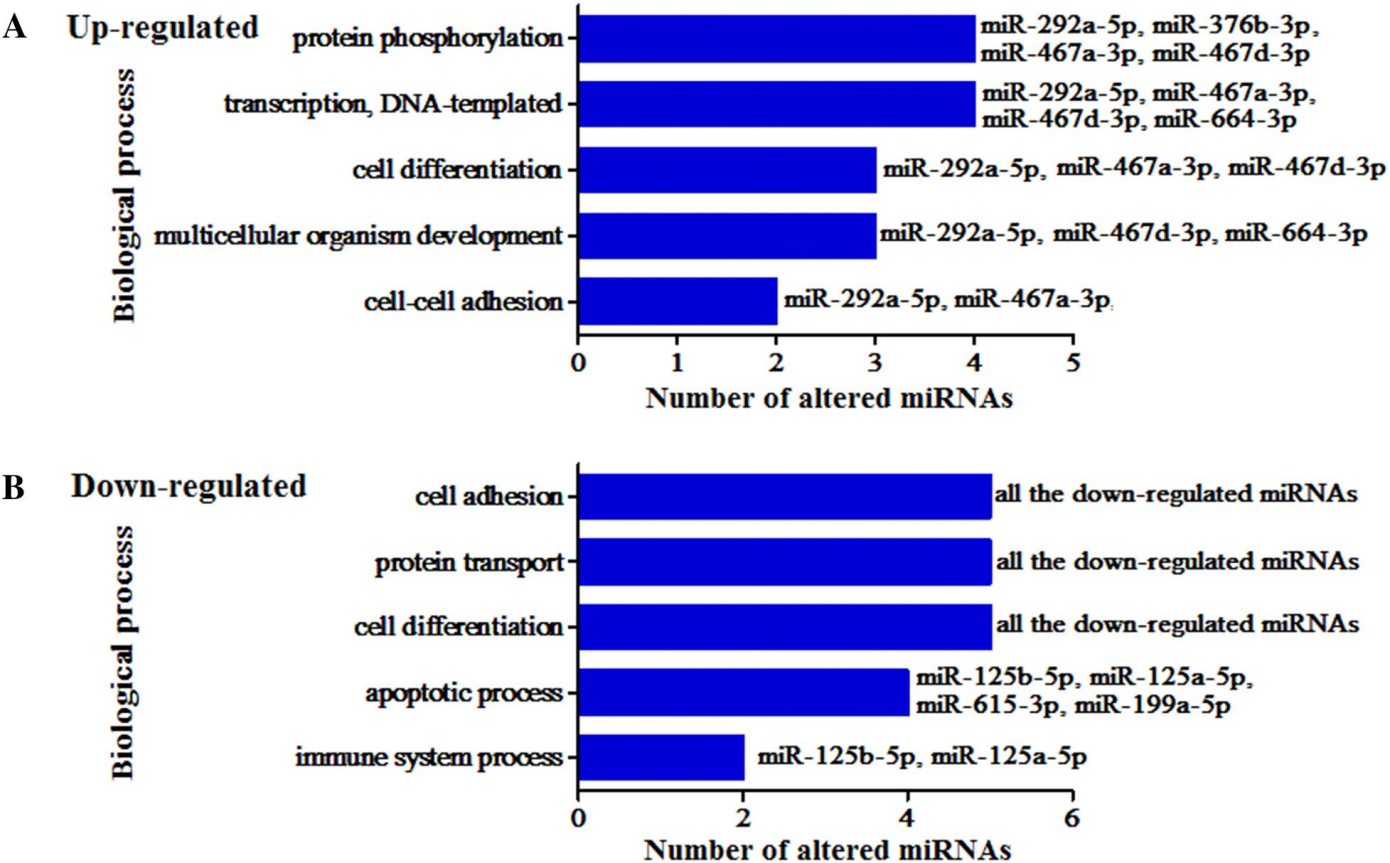
**GO analysis of target genes of altered mouse circulating miRNAs after*T. spiralis*(upregulated miRNAs for A and downregulated miRNAs for B)**. Biological process is one of the three GO categories. A full list of GO terms of the ten altered miRNAs is shown in Additional file 7.

**Figure 6:**
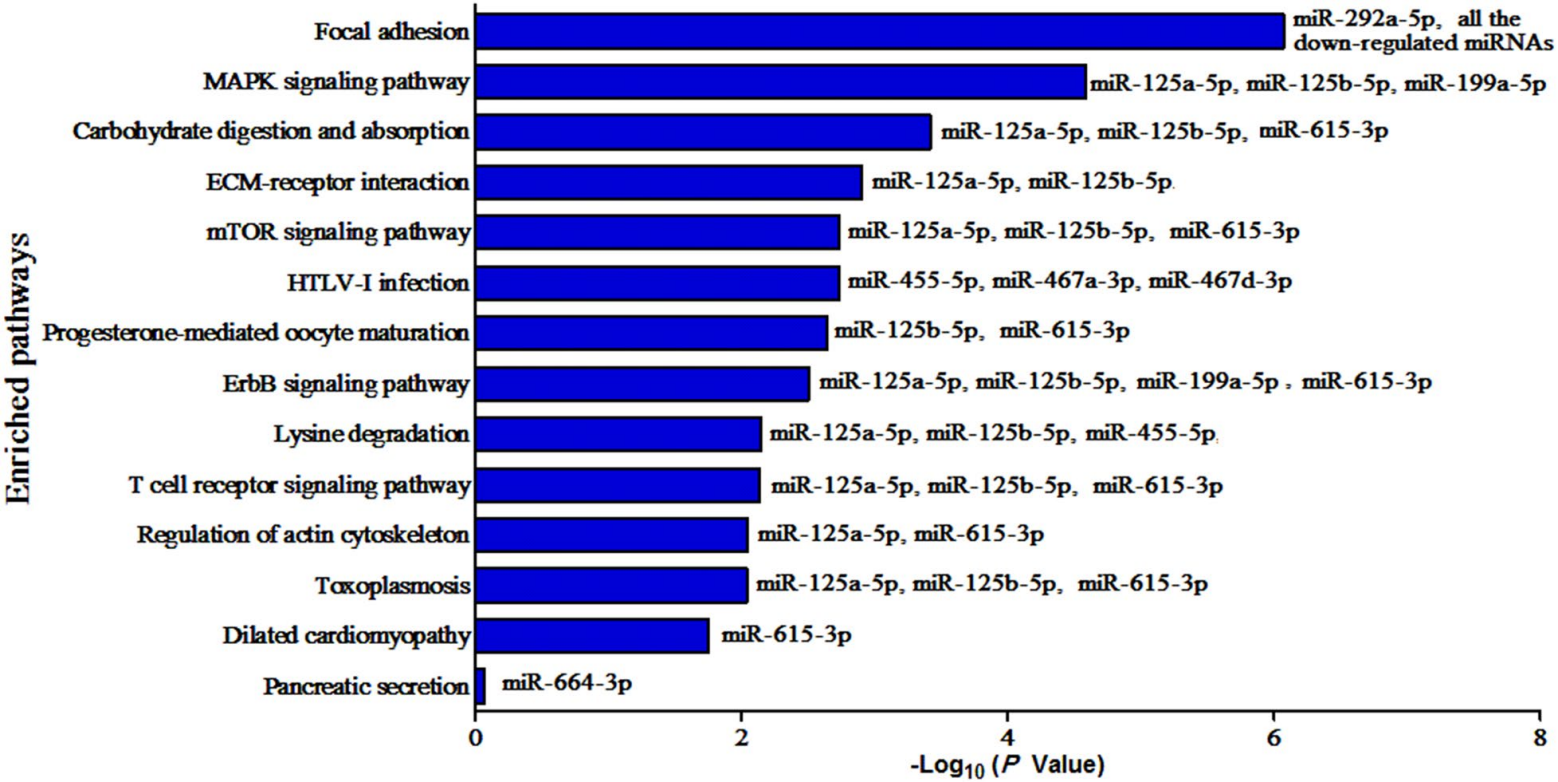
**A subset of the enriched KEGG pathways regulated by the altered mouse circulating miRNAs**. A full list of KEGG pathways is shown in Additional file 8.

**Figure 7:**
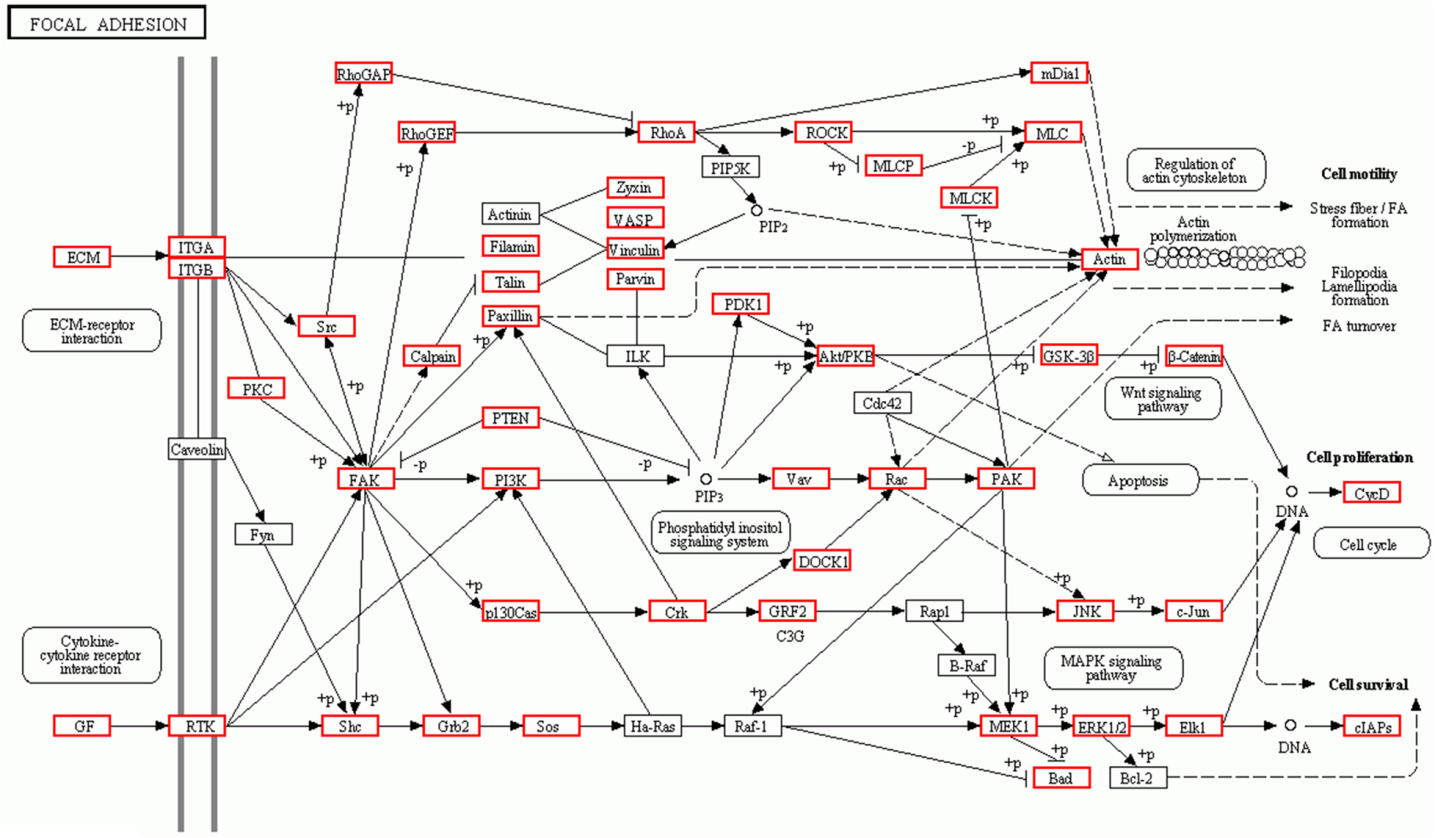
**Part of the KEGG pathway “Focal adhesion”**. Target genes of the ten differentially expressed miRNAs in mice infected with *T. spiralis* were enriched in the pathway “Focal adhesion” (red square).

To gain insights into the possible functions of differentially expressed circulating miRNAs in mice infected with *T. spiralis*, their target genes were predicted using RNAhybrid, miRanda, TargetScan, and PITA. The predicted target genes common to all four databases were considered the final miRNA target genes (Additional file 6). The number of target genes for each miRNA was 4140 for mmu-miR-125a-5p, 3092 for mmu-miR-125b-5p, 2626 for mmu-miR-199a-5p, 766 for mmu-miR-455-5p, 3392 for mmu-miR-615-3p, 522 for mmu-miR-467a-3p, 387 for mmu-miR-467d-3p, 1329 for mmu-miR-292a-5p, 191 for mmu-miR-376b-3p, and 156 for mmu-miR-664-3p. Then, the miRNA target genes were further functionally categorized into biological process (BP), molecular function (MF), and cellular component (CC) according to GO analysis (Table 2, Additional file 7). GO enrichment analysis revealed that in the BP category, the upregulated miRNAs were mainly involved in “protein phosphorylation”, “transcription, DNA-templated”, “cell differentiation”, “multicellular organism development”, and “cell–cell adhesion” (Figure 5A). With regard to the downregulated miRNAs, “cell adhesion”, “cell differentiation”, “protein transport”, “apoptotic process”, and “immune system process” were some of the most frequent GO terms (Figure 5B). KEGG pathway and enrichment analysis revealed that target genes of the differentially expressed miRNAs were involved in 297 different pathways. The enriched pathways for each miRNA are shown in Additional file 8, and part of the enriched pathways are shown in Figure 6. Target genes were mostly enriched in Focal adhesion (Figure 7), MAPK signalling pathway, Carbohydrate digestion and absorption, ECM-receptor interaction, HTLV-I infection, mTOR signalling pathway, Progesterone-mediated oocyte maturation, ErbB signalling pathway, and T cell receptor signalling pathway.

### Expression levels of the altered circulating miRNAs at different phases of infection

**Figure 8:**
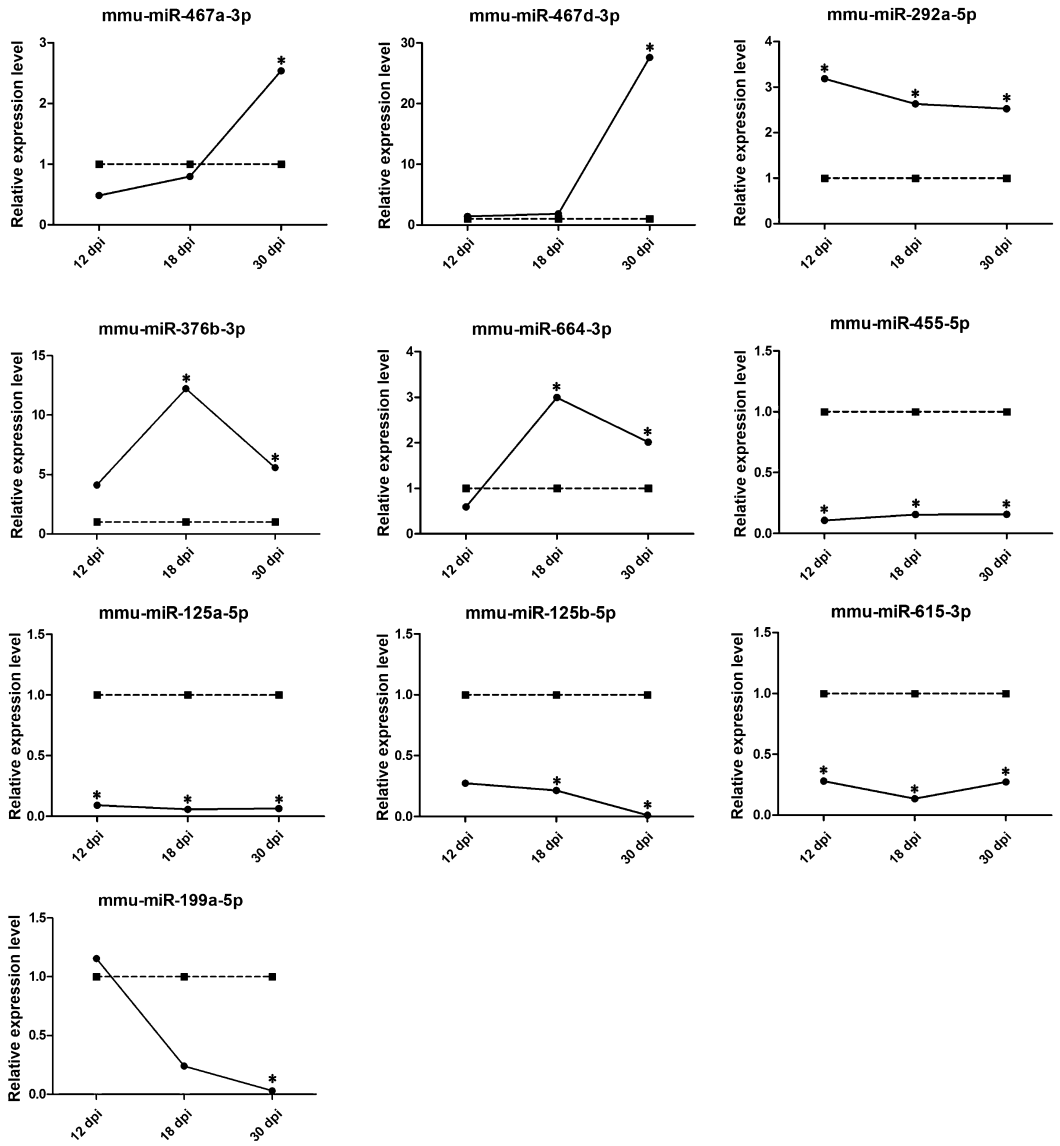
**Dynamic changes in the ten altered circulating miRNAs in mice during infection with*T. spiralis***. Expression levels were normalized to the U6 snRNA levels, and they are presented as relative expression to the control expression (mean ± SD). The relative expression levels of the controls were set as 1. *P < 0.05 was considered statistically significant.

To observe the variation in expression levels of the altered circulating miRNAs during infection, the infected sera were collected at 12, 18, and 30 dpi. The ten differentially expressed miRNAs in these serum samples were analysed by qRT-PCR. As shown in Figure 8, among the 5 upregulated miRNAs, mmu-miR-467a-3p and mmu-miR-467d-3p expression gradually increased from 12 to 30 dpi. The expression of mmu-miR-376b-3p and mmu-miR-664-3p increased significantly at 18 dpi and then decreased at 30 dpi. In addition, the expression of mmu-miR-292a-5p gradually decreased from 12 to 30 dpi. Among the 5 downregulated miRNAs, mmu-miR-199a-5p expression was significantly downregulated at 30 dpi. Interestingly, the expression levels of the other four miRNAs (mmu-miR-455-5p, mmu-miR-125b-5p, mmu-miR-125a-5p, and mmu-miR-615-3p) were significantly lower than those in the control group, showing a steady downregulation at different phases of infection.

## Discussion

It is well known that circulating miRNAs of mammalian hosts are dysregulated during helminthic infections, suggesting their regulatory roles in host-parasite interactions. Previous studies have shown that *Echinococcus granulosus* and *S. japonicum* infection could alter the expression levels of host circulating miRNAs [24, 25]. To date, no studies have investigated the profile of host circulating miRNAs during infection with *T. spiralis*. It is well known that newborn larvae released by adults burst into blood, reach skeletal muscle, and invade skeletal muscle fibres. During this process, pathological changes, such as the increase in inflammatory mediators, occur in the host serum [26]. This suggests that the host-*Trichinella* interaction could induce changes in host circulating miRNAs. Hence, in the present study, for the first time, mouse miRNAs in serum were identified at 30 dpi by high-throughput sequencing. Since the parasite at 30 dpi is in the early ML phase and newborn larvae are still continuously released by adults, at this time point worms at different developmental stages exist simultaneously: adult worms in intestine tissues, newborn larvae in blood, and pre-encapsulated larvae and ML in muscle tissues. This means that more information on host circulating miRNAs could be acquired. In total, ten dysregulated circulating miRNAs in mice infected with *T. spiralis* at 30 dpi were found in this study. Bioinformatic analysis indicated that these miRNAs might regulate many biological functions, such as binding, catalytic activity, and molecular transducer activity. The expression of these host circulating miRNAs was further analysed at different time points (12 and 18 dpi) when the larvae were in the pre-encapsulated phase and anti-*T. spiralis* antibodies in serum could not yet be detected [27]. Because circulating miRNAs might be potential biomarkers for trichinellosis or crucial mediators of host-*Trichinella* interactions, the identification of altered circulating miRNAs in *Trichinella*-infected hosts will be beneficial to further understand the mechanisms of this disease and find promising novel biomarkers for trichinellosis.

To date, some functions of the altered miRNAs found in this study have been reported. They might play their roles by targeting specific genes and participating in signalling pathways. For example, miR-467a could inhibit neural differentiation and brain development by targeting Sox6 [28], might regulate the apoptosis of thymic lymphocytes by targeting Fas and Bax [29], and could be an indicator of severe malaria infection [30]. Moreover, miR-467d might be involved in the transformation of colonic epithelial cells by regulating MAPK, PI3K, and other pathways [31]. miR-376b could inhibit macrophage autophagy by directly targeting autophagy-related Atg5 [32] and could regulate the expression of the inflammatory factor IL-6 by targeting the NF-kappa-B inhibitor zeta and STAT3 [33]. miR-664 and miR-455-5p were proven to be associated with carcinoma cell proliferation, apoptosis, and migration by targeting diverse signalling pathways [34, 35].

To further elucidate the functions of these altered miRNAs in trichinellosis, KEGG pathway analysis was used to identify signalling pathways of their predicted target genes. The results showed that “Focal adhesion” and “MAPK signaling pathway” were two of the most enriched pathways. The Focal adhesion pathway has a wide range of biological functions, including the immune response and barrier function. Focal adhesion, acting as a cellular sensor and regulator, could anchor cells to the extracellular matrix and directly assemble the prestressed actin cytoskeleton [36]. Previous studies have shown that the inhibition of focal adhesion formation facilitates the hypermotility of monocytes infected with *Toxoplasma gondii* (*T. gondii*) [37], and the dysregulation of focal adhesion kinase facilitates the transmigration of *T. gondii* across polarized endothelial cell monolayers [38]. Moreover, the loss of focal adhesion kinase in endothelial cells could enhance cell attachment and cell–cell contacts [39]. Focal adhesion could also promote the migration of neutrophils from blood vessels to infection sites by participating in endothelial conformational changes [40]. In the present study, an enrichment of the Focal adhesion pathway was found in *T. spiralis* infection, and GO analysis of the predicted target genes identified the GO term “cytoskeletal protein binding and endomembrane system”. These results suggest that the expression of genes related to focal adhesion might be altered in hosts infected with *T. spiralis*, resulting in changes in focal adhesion and tight junction formation of epithelial monolayers, which might be beneficial for host defences against *T. spiralis* infection. Among the altered miRNAs, mmu-miR-125a-5p and mmu-miR-615-3p might be involved in the Focal adhesion pathway by targeting Pik3cd, laminin or other genes.

The mitogen–activated protein kinase (MAPK) pathway, which includes p38, extracellular signal-related kinase (ERK), and Jun amino-terminal kinase (JNK1/2/3), plays an important role in cell proliferation, differentiation, migration, senescence, and apoptosis [41]. In addition, the MAPK signalling pathway also participates in innate and acquired immune responses during parasitic infection. For example, macrophages can produce IL-33 upon activation of the MAPK signalling pathway, and IL-33 can facilitate the Th2 immune response by inducing the production of proinflammatory factors and Th2-associated cytokines and activating lymphocytes, eosinophils and mastocytes [42]. Previous studies have proven the functions of IL-33 against infection with various parasites, including *Schistosoma japonica*, *Angiostrongylus*, *Trichuris muris*, and hookworm [43, 44]. In addition, the MAPK pathway could also downregulate proinflammatory factors by targeting miR-664 in the ileum [45]. In this study, target genes (such as MAPK1) of miR-664 were enriched in the MAPK pathway, suggesting that the alteration of miR-664 would regulate proinflammatory factors through the MAPK pathway in hosts infected with *T. spiralis*. These findings were consistent with the enriched GO term “activation of MAPK activity”. Moreover, miR-376 is a mediator of the MAPK pathway [46], and miR-125 could decrease the phosphorylation of MAPK pathway component, including ERK, p38, and JNK [47]. This study showed that mmu-miR-376b-3p, mmu-miR-664-3p, mmu-miR-125b-5p, and mmu-miR-125a-5p were involved in the MAPK pathway, supporting the hypothesis that the MAPK pathway might participate in the dysregulation of pathological responses during *T. spiralis* infection.

Host immune responses to *T. spiralis* infection mainly occur in the intestinal phase and muscular phase [48]. Invasion by ML can result in host muscle damage and an intensive inflammatory response, a complex T-helper type-2 (Th2) immune response. The Th2 cytokine IL-10 has been proven to be an important regulator during host-parasite interactions. It can limit the inflammatory responses and protect the host against injury during the acute phase of muscle infection while inducing the Th2 response to protect the mature, infectious parasite during chronic infection [49]. The production of IL-10 requires the phosphorylation of MAPK, which was one of the most enriched pathways in this study [50]. Among the ten miRNAs, the downregulated mmu-miR-125a-5p, mmu-miR-125b-5p, and mmu-miR-615-3p were enriched in the MAPK pathway by targeting the tumour necrosis factor receptor superfamily (Tnfrsf1a), tumour necrosis factor (Tnf), or dual specificity phosphatase (Dusp 4, 6, 7, and 8). In addition, mmu-miR-125a-5p, mmu-miR-125b-5p, and mmu-miR-199a-5p were found to target IL-10 receptor alpha (IL10RA), suggesting that these miRNAs might affect the functions of IL-10 to then regulate the Th2 immune response during *T. spiralis* infection.

*T. spiralis* infection results in alterations of small intestinal physiology, which includes changes in mucus production and intestinal motility, and initiates immune responses mediated by cytokines released from Th2 cells [51]. A potent Th2-type immune response could induce intestinal muscle hypercontractility and worm expulsion during the intestinal phase. Attenuated muscle contractility and reduced parasite expulsion were found in athymic mice infected with *T. spiralis* [52], suggesting that the host T cell-mediated immune response plays a leading role in parasite expulsion. Th2-type cytokines, such as IL-4 and IL-13, can promote *T. spiralis* expulsion by T cells and mast cells [53]. Moreover, Th1 responses are also involved in immune regulation during parasite infection and suppress effective anti-parasite immunity. Th1-type cytokines, such as IL-12 and IFN-γ, can inhibit Th2-type immune responses by inhibiting IL-4 production [54]. In addition, the CD40-CD40 ligand interaction influences the production of monocyte chemoattractant protein-1 (MCP-1), whose deficiency results in a shift from Th2 to Th1 immune responses [55, 56]. In this study, the downregulated mmu-miR-615-3p and mmu-miR-455-5p targeted CD40, CD40 ligand (CD40LG), IFN-γ receptor 2 (IFNGR2), or interleukin 12B (IL-12B). It could be speculated that these downregulated host miRNAs might regulate the intensity of the Th2 immune response induced by *T. spiralis* infection and promote parasite expulsion.

Mucins, the protective barrier of the underlying epithelium, play a protective role against infection by trapping parasites and inhibiting parasite motility and feeding capacity. The ingestion of mucus by the worms may have a deleterious effects on them. In the small intestine, excess production of mucus by hyperplastic goblet cells has been found in infections with multiple parasites [51]. In addition, mucin genes were found to be upregulated during parasite infections, such as *T. spiralis* infection [57]. Cytokines, especially Th2-type cytokines (IL-4, IL-9, and IL-13), can regulate mucin production. They can induce goblet cell hyperplasia and mucin production by Stat6 pathways during *T. spiralis* infection. Otherwise, laminins are the major structural component of the basement membrane, which is a meshwork of proteins separating the epithelium from connective tissue. Laminin can enhance myotube formation by modulating type I collagen films after damage to skeletal muscle [58]. In the present study, functional analyses showed that all five downregulated miRNAs might target laminin, suggesting that they could regulate the expression of laminin and promote repair of host skeletal muscle injury caused by *T. spiralis* infection.

Since the profiles of circulating miRNAs could be changed in host serum during parasite infection, characterizing these profiles would be helpful in understanding the roles of miRNAs in the regulation of molecular networks related to infection. Therefore, the roles of circulating miRNAs (especially upregulated miRNAs) as potential biomarkers of parasitic diseases have become increasingly evident [29]. This work further investigated the expression levels of the ten altered circulating miRNAs at 12, 18, and 30 dpi. The results showed that among the 5 upregulated miRNAs, mmu-miR-467a-3p and mmu-miR-467d-3p expression reached a peak at 30 dpi, while the expression of mmu-miR-376b-3p and mmu-miR-664-3p increased significantly at 18 dpi and then decreased at 30 dpi. In addition, the expression of mmu-miR-292a-5p gradually decreased from 12 to 30 dpi. The variation of individual miRNAs at different time points might be associated with the infection phases of *T. spiralis*; hence, detailed detection of the kinetics of these altered circulating miRNAs could reveal novel candidate biomarkers for trichinellosis, especially in the early phase of infection. Certainly, the clinical potential of these altered miRNAs needs to be verified in further studies.

In conclusion, this study provides the profiles of differentially expressed circulating host miRNAs during *T. spiralis* infection. GO and KEGG pathway enrichment analyses were performed for a functional analysis of the target genes of the ten altered miRNAs. In particular, the pathways “Focal adhesion” and “MAPK signaling pathway” were enriched during *T. spiralis* infection and might be important mediators in the host-*Trichinella* interactions. This paper will be helpful in understanding the mechanisms involved in *Trichinella* parasitism and provide promising serum biomarkers for trichinellosis.

## Supplementary information

**Additional file 1. Overview of small RNA-seq data of all libraries**.

**Additional file 2. Distribution of the different types of small RNA**.

**Additional file 3. The sequences of known miRNAs in*T. spiralis*miRNA libraries**.

**Additional file 4. The sequences of novel miRNAs in*T. spiralis*miRNA libraries**.

**Additional file 5. The mean expression levels of miRNAs in the sera of the infected and uninfected mice**.

**Additional file 6. The predicted target genes of each altered circulating miRNA in mice infected with*T. spiralis***.

**Additional file 7. GO analysis of target genes of each altered circulating miRNA in mice infected with*T. spiralis***.

**Additional file 8. KEGG analysis of target genes of each altered circulating miRNA in mice infected with*T. spiralis***.

## Data Availability

The datasets used and/or analysed during the current study are available from the corresponding author upon reasonable request.
