# Supplementary material for: Comparative expression profiles of host circulating miRNAs in response to Trichinella spiralis infection
Source: Vet Res. 2020 Mar 11;51:39. doi: 10.1186/s13567-020-00758-0 (PMC7065375; doi:10.1186/s13567-020-00758-0)
Supplement: Supplementary file 1 — Additional file 1. Overview of small RNA-seq data of all libraries. [file 13567_2020_758_MOESM1_ESM.doc]

**Additional file 1 Overview of small RNA seq data of all libraries**

|  | Infected group (E) | | |  | Uninfected group (C) | | |
| --- | --- | --- | --- | --- | --- | --- | --- |
| Type | Reads number (E1) | Reads number (E2) | Reads number (E3) |  | Reads number (C1) | Reads number (C2) | Reads number (C3) |
| Total reads | 30333057 | 28252287 | 37716729 |  | 36760408 | 36484242 | 36546692 |
| High quality | 30106516 | 27723286 | 36995457 |  | 36054794 | 35779051 | 35841701 |
| 3′ adapter | 2169303 | 3930511 | 10423276 |  | 7036481 | 6005061 | 5443395 |
| Insert | 76082 | 189575 | 2443994 |  | 1203176 | 2189861 | 1562649 |
| 5′ adapter | 739368 | 623535 | 3983065 |  | 2400509 | 1357217 | 1482647 |
| Below 18 nt | 2166232 | 2326211 | 2166977 |  | 2410552 | 1489583 | 1809995 |
| PolyA | 20 | 35 | 43 |  | 31 | 160 | 137 |
| Clean reads | 24955511 | 20653419 | 17978102 |  | 23004045 | 24737169 | 25542878 |
