# Supplementary figures and images for: Comparative expression profiles of host circulating miRNAs in response to Trichinella spiralis infection
Source: Vet Res. 2020 Mar 11;51:39. doi: 10.1186/s13567-020-00758-0 (PMC7065375; doi:10.1186/s13567-020-00758-0)

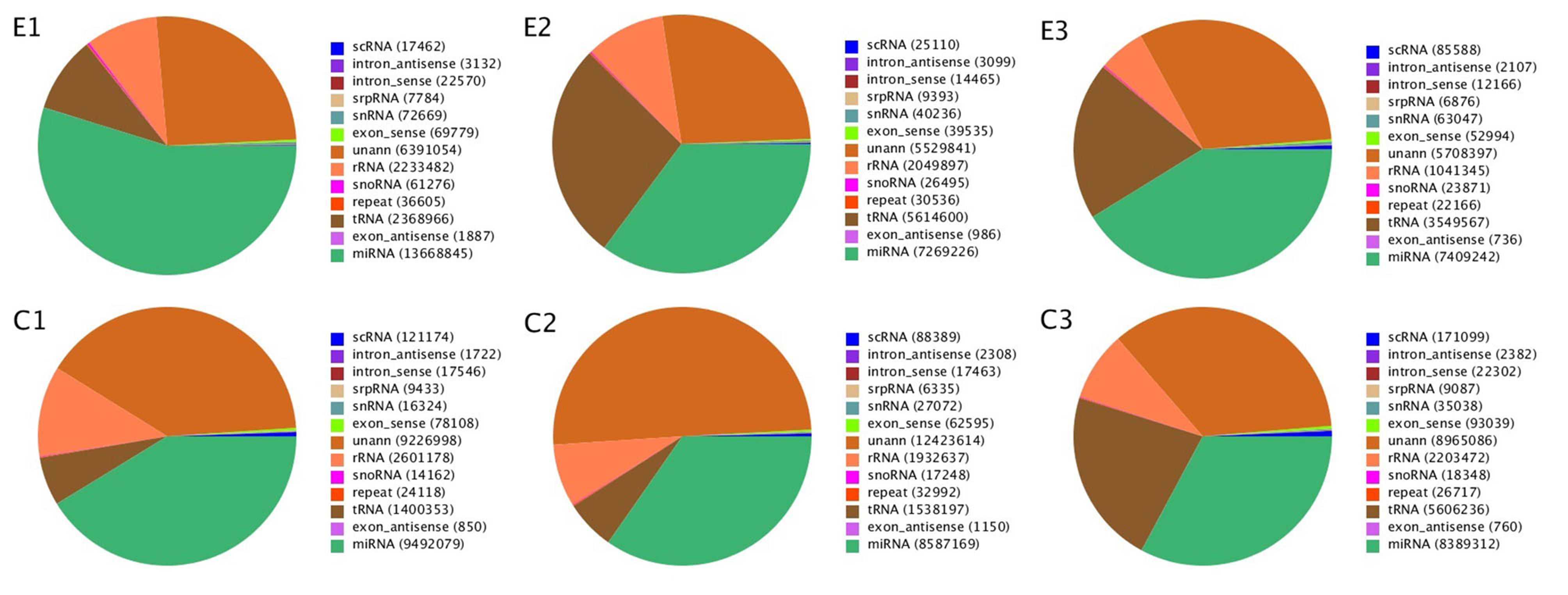

Supplement: Supplementary file 2 — Additional file 2. Distribution of the different types of small RNA. [file 13567_2020_758_MOESM2_ESM.jpg]
